# Combined Use of MITRACLIP and Ventricular ASSIST Devices in Cardiogenic Shock: MITRA-ASSIST Registry

**DOI:** 10.3390/jcm13154408

**Published:** 2024-07-28

**Authors:** Borja Rivero-Santana, Alfonso Jurado-Roman, Isaac Pascual, Chi Hion Li, Pilar Jimenez, Rodrigo Estevez-Loureiro, Pedro Cepas-Guillén, Tomás Benito-González, Ana Serrador, Jose Maria De La Torre-Hernandez, Pablo Avanzas, Estefania Fernandez-Peregrina, Luis Nombela, Berenice Caneiro-Queija, Xavier Freixas, Felipe Fernandez-Vazquez, Ignacio Amat-Santos, Dae-Hyun Lee, Victor Leon, Dabit Arzamendi, Raul Moreno, Guillermo Galeote

**Affiliations:** 1 Cardiology Department, La Paz University Hospital, 28046 Madrid, Spain; raulmorenog@hotmail.com (R.M.); ggaleote1@gmail.com (G.G.); 2 Hospital La Paz Institute for Health Research, IdiPAZ, 28029 Madrid, Spain; 3 Hospital Universitario Central de Asturias, 33011 Oviedo, Spainavanzas@secardiologia.es (P.A.); citasconsultas2.gae@sespa.es (V.L.); 4 Hospital de la Santa Creu i Sant Pau, 08025 Barcelona, Spain; ch.pedroli@gmail.com (C.H.L.); efernandezpe@santpau.cat (E.F.-P.); darzamendi@santpau.cat (D.A.); 5 Hospital Clínico San Carlos, 28040 Madrid, Spain; patropjq@gmail.com (P.J.); luisnombela@yahoo.es (L.N.); 6 Complexo Hospitalario Universitario de Vigo, 36312 Vigo, Spain; roiestevez@hotmail.com (R.E.-L.); bcanque@gmail.com (B.C.-Q.); 7 Hospital Clinic, 08036 Barcelona, Spain; pedro.cepasguillen@gmail.com (P.C.-G.); freixa@clinic.cat (X.F.); 8 Hospital Universitario de León, 24008 León, Spain; tomasbenito@outlook.com (T.B.-G.); atepac.hlo@saludcastillayleon.es (F.F.-V.); 9 Hospital Clínico de Valladolid, 47003 Valladolid, Spain; aserradorf@gmail.com (A.S.); ijamat@gmail.com (I.A.-S.); 10 Hospital Universitario Marqués de Valdecilla, 39008 Santander, Spain; chematorre60@gmail.com (J.M.D.L.T.-H.); daehyun.lee@scsalud.es (D.-H.L.)

**Keywords:** cardiogenic shock, mitral regurgitation, transcatheter edge-to-edge repair, mechanical circulatory support, heart failure

## Abstract

**Background:** Patients with cardiogenic shock (CS) and mitral regurgitation (MI) have a prohibitive risk that contraindicates surgical treatment. Although the feasibility of transcatheter edge-to-edge therapy (TEER) has been demonstrated in this setting, the benefit of the combined use of TEER with mechanical circulatory support devices (MCS) has not been studied. The aim of this study was to evaluate the clinical outcomes of TEER in patients with MCS. **Methods:** The MITRA-ASSIST study is a retrospective multicentre Spanish registry that included patients with MR and CS who underwent TEER in combination with MCS. The primary endpoint was death from any cause at 12 months. The secondary endpoint was a composite of death from any cause or hospitalisation for heart failure at 12 months. **Results:** A total of twenty-four patients in nine high-volume Spanish centres (66.2 (51–82) years, 70.8% female, EuroSCORE II 20.4 ± 17.8) were included. Acute ST-elevation myocardial infarction was the main CS aetiology (56%), and the most implanted MCS was the intra-aortic balloon pump (82.6%), followed by ECMO (8.7%), IMPELLACP^®^ (4.3%), or a combination of both (4.3%). Procedural success was 95.8%, with 87.5% in-hospital survival. At 12-month follow-up, 25.0% of patients died, and 33.3% had a composite event of death from any cause or hospitalisation for heart failure. **Conclusions:** TEER in patients with concomitant CS and MR who require MCS appears to be a promising therapeutic alternative with a high device procedural success rate and acceptable mortality and heart failure readmission rates at follow-up.

## 1. Introduction

Cardiogenic shock (CS) continues to have an unfavourable prognosis despite the use of all currently available treatment options. Even when patients are adequately treated, the 30-day mortality rate may often exceed 50% [1,2]. In addition, CS with concurrent valvular heart disease is common and aggravates the prognosis of these patients. Several studies have shown that moderate-to-severe mitral regurgitation (MR) is present in approximately 10–20% of patients with CS [3,4] and significantly increases the risk of death by up to three times [5,6].

Surgical intervention for mitral valve disease may have benefits in the setting of CS. However, it is generally not a viable option due to the prohibitive risk associated with most cases of CS and MR [7]. Consequently, transcatheter edge-to-edge repair (TEER) has emerged as a potential therapeutic alternative [8,9,10]. To date, many studies have shown that TEER is a feasible procedure for CS and can reduce mortality and heart failure readmissions.

However, despite the promising results, more than 20% of patients with CS require mechanical ventricular support (MCS) for hemodynamic support, and the feasibility and benefit of TEER in this setting have not been established [11,12]. To date, the combined use of MCS and TEER in patients with CS and MR is marginal because the evidence is limited to case series [13,14]. The MITRA-ASSIST Registry is the first study specifically designed to evaluate the use of TEER in CS and MR patients requiring MCS.

## 2. Materials and Methods

### 2.1. Study Population

The MITRA-ASSIST Registry is a multicentre retrospective registry that included patients with grade 3+ (moderate to severe) or 4+ (severe) MR and CS who underwent TEER in combination with MCS between June 2016 and December 2022 at nine Spanish high-volume centres. Only patients with Society for Cardiovascular Angiography and Interventions (SCAI) stage C to E cardiogenic shock were selected. Both functional mitral regurgitation and degenerative mitral regurgitation were included, and there were no specific anatomic requirements except the technical feasibility of grasping the mitral leaflets. All patients were found to be eligible for TEER by the Heart Team at each centre after being deemed unsuitable for other therapies, such as mitral valve surgery, heart transplantation, or implantation of a permanent left ventricular assist device (LVAD). The study was approved by the ethics committees of the participating hospitals.

### 2.2. Definitions

Cardiogenic shock (CS) was defined as hypotension (SBP < 90 mmHg) despite adequate filling status with signs of hypoperfusion according to the diagnostic criteria used in the European Guidelines for the diagnosis and treatment of acute and chronic heart failure [15]. The following signs of hypoperfusion were considered: cold extremities, oliguria, altered mental status (AMS), narrow pulse pressure, metabolic acidosis, elevated serum lactate (≥2.5 mmol/L), and elevated serum creatinine.

Functional mitral regurgitation (FMR) was defined as regurgitation produced through the mitral valve in the absence of organic involvement of the elements that integrate the valvular and subvalvular apparatus. Degenerative mitral regurgitation (DMR) was defined as mitral regurgitation resulting from primary anatomical alterations of the mitral valve apparatus.

### 2.3. Procedure

**Figure 1:**
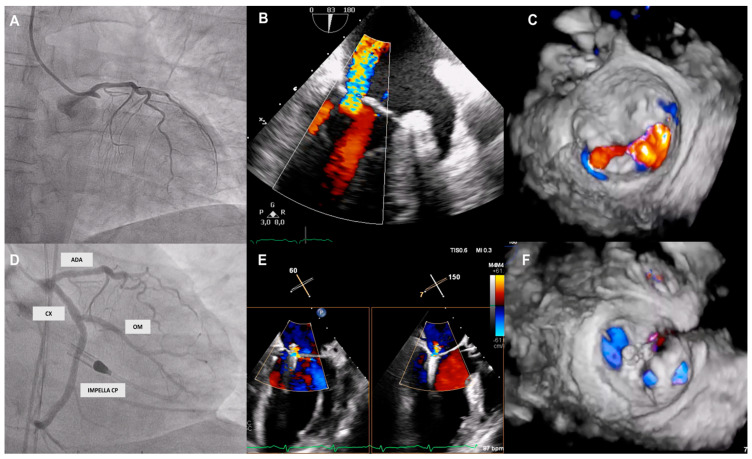
Multimodal imaging of a MitraClip implantation in a patient with IMPELLA CP^®^ device support. Angiography shows acute myocardial infarction due to occlusion of the proximal circumflex artery (**A**) with severe functional ischemic mitral regurgitation. On transoesophageal echocardiography (TEE) (**B**) and 3D reconstruction (**C**), a mid-level jet was observed, predominantly between P2-A2. (**D**). Angiography confirms the correct placement of IMPELLA CP^®^ device and the optimal results of the previously implanted stents. The valve was repaired by implantation of a MitraClip NTW device at A3-P3 and a second NT clip at A2-P2, with excellent results as assessed by TEE (**E**) and 3D reconstruction (**F**). ADA: anterior descendent artery; CX: circumflex; OM: obtuse marginal.

The procedure was performed in the catheterization room under general anaesthesia, using a 24F guiding catheter from the femoral vein and guided by fluoroscopy and transoesophageal echocardiography (TEE). In all cases, TEER was performed with the third (NTR and XTR) and fourth (NT, NTW, XT, and XTW) generation devices of the MitraClip^®^ system (Abbott Vascular, Santa Clara, CA, USA). Acute success of the procedure was defined as MR reduction of ≥1 grade and a final MR to grade ≤ 2+ after placement of at least one clip with a transmitral gradient (TPMG) < 5 mm Hg. In all cases, the hemodynamic support of the ventricular assist devices had to be reduced to the minimum allowed according to the clinical situation of the patient to adequately assess the TPMG and the degree of residual MR, both after the first clip and to assess the result. Furthermore, in those patients who were hemodynamically supported by the IMPELLA^®^ device (Abiomed, Danvers, MA, USA), TEE was used to monitor the possibility of punctual interference of the MitraClip^®^ release catheter with the IMPELLA^®^ catheter when crossing the mitral valve to capture the leaflets (Figure 1).

### 2.4. Echocardiographic Evaluation

Echocardiographic measurements were conducted prior to, during, and after the procedure and before discharge. The severity of mitral regurgitation was graded according to the criteria recommended in the European guidelines for the management of valvular heart disease [15]. Additional echocardiographic parameters such as left ventricular ejection fraction (LVEF), left ventricular end-diastolic diameter (LVEDD), tricuspid annular plane systolic excursion (TAPSE), effective regurgitant orifice area (EROA), TMPG, and the presence of a ruptured mitral chord or prolapse were collected.

### 2.5. Outcomes

The primary endpoint was death from any cause at 12 months. The secondary endpoint was a composite event of death from any cause or hospitalisation for HF at 12 months. In addition, in-hospital complications such as minor and major bleeding, acute kidney injury (AKI), myocardial infarction (MI), and acute stroke were collected. Bleeding events and vascular complications that were procedure-related were defined using Bleeding Academic Research Consortium (BARC) definitions. AKI was defined according to Kidney Disease: Improving Global Outcomes (KDIGO) criteria as any of the following: increase in serum creatine (SCr) by 0.3 mg/dL or more within 48 h; increase in SCr baseline to 1.5 times or more, which is known or presumed to have occurred within the prior 7 days; or urine volume < 0.5 mL/kg/h for 6 h [16]. MI was defined as the development of recurrent angina symptoms accompanied by changes in the 12-lead electrocardiogram or increased levels of cardiac-specific biomarkers. A stroke was defined as an acute episode of focal neurological dysfunction that persists for more than 24 h.

### 2.6. Statistical Analysis

Variables were expressed as mean and standard deviation (SD) or as median and interquartile range (IQR), where appropriate. The normality of the distribution of continuous variables was explored using the Shapiro–Wilk tests. Categorical variables were presented as counts and percentages and were compared. Differences between all groups were studied with ANOVA analysis for continuous variables and using the chi-square or Fisher exact test for categorical data. The survival rate free from clinical endpoints was estimated by the Kaplan–Meier method and compared by a log-rank test. An overall alpha level of 0.05 was used as the cut-off point for statistical significance, and all statistical tests were two-tailed. All data was analysed with R Project 4.2.1 (R Foundation for Statistical Computing, Vienna, Austria).

## 3. Results

### 3.1. Patient Characteristics

A total of 24 patients were included. Baseline characteristics of the study population are given in Table 1. The mean age of the patients was 65.7 ± 8.7 years; 70.8% were female; 50% had a previous infarction; and 20.8% had previous MR. The mean EuroSCORE II risk score was 20.4 ± 17.8, and the STS risk score was 12.4 ± 10.1.

### 3.2. Shock Characteristics

Shock characteristics are presented in Table 1 and Table 2. The most frequent cause of CS was acute ST-elevation myocardial infarction (54.2%), with the circumflex coronary artery being the culprit vessel in most cases (circumflex coronary artery 41.6%, right coronary artery 26%, other vessels 8.4%). At the time of admission, most patients were classified as INTERMACS class 2 or 3 (75%), and SCAI C (54.2%), with 66.7% experiencing multiple organ failure. Prior to TEER therapy, 45.8% of patients required invasive mechanical ventilation, 16.7% required continuous renal replacement therapy (CRRT), and 4.2% had suffered a cardiac arrest. The mean time from the onset of shock to MCS implantation was 5.9 ± 4.2 days. Inotropic support was used in 15 patients (62.5%) and vasopressor treatment in 18 patients (75%). The mean arterial blood pressure was 52.5 ± 8.2 mm Hg, and the mean pulmonary capillary wedge pressure was 22.5 ± 5.3 mm Hg. The additional hemodynamics and laboratory characteristics prior to TEER therapy are detailed in Table 2.

### 3.3. Echocardiographic Characteristics

Baseline echocardiographic measurements are given in Table 3. The mean LVEF was 33.4 ± 9.6%, the left ventricular end-diastolic diameter (LVEDD) was 5.5 ± 0.9 cm, and the TAPSE was 17.4 ± 2.9 cm. Regarding mitral regurgitation, 83.3% of patients had FMR, while 16.7% had DMR. MR was graded as 4+ and 3 in 95.8% and 4.2% of patients, respectively. The mean EROA was 0.7 (±0.5) cm^2^ by PISA evaluation. In three patients (15%), there was a rupture of the mitral chord. LVEF, LVEDD, EROA, TAPSE, and colour Doppler MR were obtained in all the cases.

### 3.4. Procedural Features and In-Hospital Outcomes

Procedural characteristics are depicted in Table 4. The mean time from the onset of shock to clip implantation was 8.8 ± 19.6 days. In the STEMI subgroup, the mean time was 7 ± 7.9 days. Procedural success was achieved in 95.8% of cases, and the mean number of clips implanted was 1.6 ± 0.8. The mean procedure time was 95.6 (61–126) min, and the post-procedural MV mean gradient was 3.1 ± 1.2 mmHg.

Data regarding clinical evolution after the procedure are shown in Table 4. MCS could be removed in 83.4% of patients in 1.7 ± 1.8 days after the TEER procedure. The duration of ICU and hospital stays was 15 (5–20) and 34 (17–36) days, respectively. Severe procedure-related major bleeding (BARC > 2) occurred in 12.5% of cases, and 29.2% of patients met AKI criteria. No MI or stroke was reported during the hospital outcome.

In-hospital mortality was 12.5%. A total of three patients died, although the TEER procedure was successful for all of them. The reported causes of death were cardiovascular in two patients and infectious diseases in one patient. At discharge, the severity of residual MR persisted at ≤2 in all cases. Of the patients who were discharged home, all were able to be discharged without the need for additional circulatory support therapies, with the exception of one patient who required LVAD implantation.

### 3.5. Mechanical Circulatory Support

All patients were supported by MCS prior to the TEER procedure. The intra-aortic balloon pump was the most frequently implanted MCS (79.2%), followed by ECMO (8.3%), IMPELLA CP^®^ (8.3%), or the combination IMPELLA CP^®^ and ECMO (4.2%). TEER was carried out successfully independently of the type of MCS. There were also no differences in the rate of complications, days of admission to the ICU, days of hospitalisation, or death during hospitalisation according to the type of ventricular assist device implanted (Table 5).

### 3.6. Outcomes

**Figure 2:**
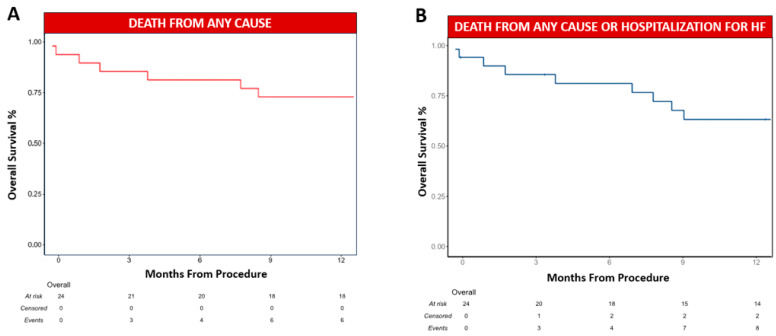
The 12-month Kaplan–Meier survival curves for death from any cause (**A**) and the composite of death from any cause or hospitalisation for heart failure at 12 months (**B**). Vertical dashes indicate censored data. HF: heart failure.

**Figure 3:**
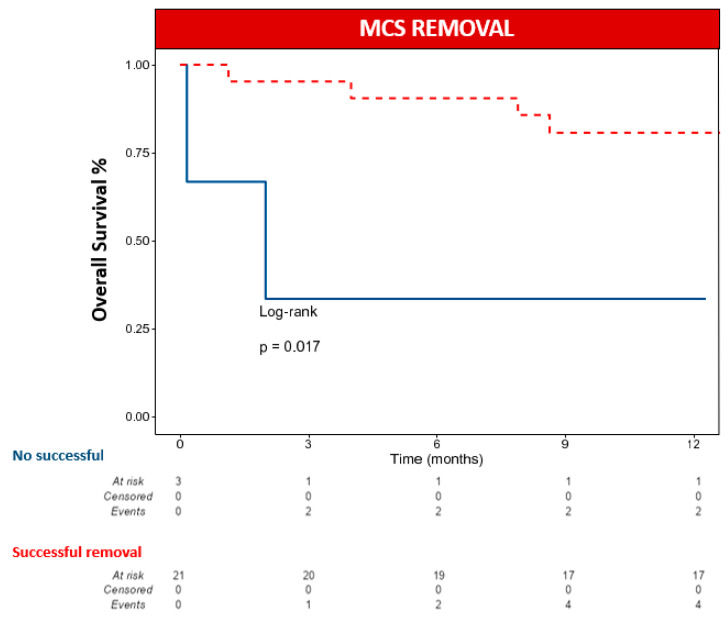
The 12-month Kaplan–Meier survival curves for all-cause mortality according to the possibility of MCS removal. MCS: mechanical circulatory support.

The median follow-up was 414 days (IQR 343–1236). At 12-month follow-up, six patients died (25.0%), and the reported causes of death were cardiovascular in four patients, infectious disease in one patient, and cancer in one patient (Table 5 and Figure 2A). At 12-month follow-up, eight patients had a composite event of death from any cause or hospitalisation for HF (Table 5 and Figure 2B). MCS could not be removed after clip implantation in three patients. However, in one case, the patient could be discharged from the hospital after upgrading to long-term ventricular support. Heart transplantation was not performed in any case. MCS removal was associated with lower death rates from any cause at 12 months, according to a univariate analysis of the Cox regression model. Kaplan–Meier survival curves showed that patients who could not be weaned from MCS had a higher mortality rate (Figure 3).

A comparative analysis was performed of the characteristics of those patients who died compared to those who survived (Appendix A Table A1). Differences were only found in age. The patients who died were significantly older than those who survived (73.6 ± 5.7 vs. 62.5 ± 7.7; *p* < 0.002).

## 4. Discussion

The Mitra-ASSIST registry is the first study to specifically explore TEER treatment in CS patients requiring MCS. The main findings of this study are that the success rate of the intervention is greater than 95%, allowing MCS removal in most patients with a low complication rate and an acceptable rate of mortality and readmissions for HF at short and 12-month follow-up.

Mortality in cardiogenic shock remains very high despite all therapeutic advances [4,17,18]. In addition, it should be noted that CS with concomitant valvular disease is present in up to 20% of cases [8,19]. These patients are underrepresented in clinical studies, constituting a management challenge. Although surgery could be an option, due to the prohibitive surgical risk of these patients, surgical treatment is often not feasible [17]. For this reason, TEER has been used in recent years as a potential therapeutic alternative. Although the evidence is limited and is confined to several case series and multicentre registries, the results are promising [8,9,20]. In a recent observational study involving 3797 patients with CS undergoing TEER, the procedural success was 85.6%, with a 48% reduction in 1-year mortality and a 46% reduction in HF readmission at 1 year in those patients who achieved an MR ≤ 2+ grade [21].

Despite these promising results, the use of TEER therapy in patients with CS requiring MCS for hemodynamic support is poorly studied. MCS in the setting of CS with MR may provide benefits by reducing preload, increasing cardiac output, and, depending on the implanted support, improving coronary percussion. However, it is not known whether the combination of TEER therapy in patients with CS and MCS may be useful. To date, the only study performed specifically in this setting is a case series involving six patients who underwent a combined MITRACLIP^®^ and IMPELLA^®^ implantation procedure [13]. The procedure was successfully performed in all cases and was associated with a hospital survival rate of 83%. However, this study has not proven benefits beyond the first 6 months, nor did it include other types of MCS different from IMPELLA^®^.

It is important to note that the use of any type of CSM was allowed for the analysis. Most patients received IABP (79.2%), followed by ECMO (8.3%), IMPELLA (8.3%), or a combination of both (4.3%). A favourable conclusion of our study is that the procedure had high effectiveness without differences in in-hospital mortality or in the rate of complications independently of the implanted MCS. In addition, in the MITRA-ASSIST registry, the procedural success was very high (95.6%), the procedure time was shorter than expected compared to previous series (95.6 (61–126) min), and the MR at discharge was less than two in all cases. These are good results compared to contemporary studies in which the success rate was similar but the implantation time and complication rate were higher even in patients who did not require MCS [18,19,21,22].

In terms of events, the results are promising. A total of 87.5% of patients were able to be discharged. Moreover, at 12-month follow-up, only 25.0% died, and 33% had a composite event of death from any cause or hospitalisation for HF. The MITRA-ASSIST study population possibly presented a priori a worse vital prognosis than in other published studies because all patients required MCS for adequate hemodynamic support. Even so, compared with other contemporary studies, the results are better than expected for a population that has not been studied to date.

The role of MCS in patients with CS depends on the time of onset of MR and the severity of shock [23]. When MR is present before the implant of MCS, ECMO is not recommended because it increases afterload and worsens pulmonary edema. IABP or IMPELLA, which provides ventricular unloading, are preferable in these cases [24]. However, MR is often diagnosed or develops at a late stage when an MCS has been implanted. Furthermore, even with an early diagnosis of MR, ECMO may be the only effective hemodynamic support option for patients with right ventricular dysfunction [25]. However, it is important to note that TEER under ECMO is safe and feasible, although it is advisable to confirm the severity of MR prior to the procedure. Although it is true that MR may improve after correction of the cause of shock, especially those secondary to coronary syndrome, up to 50% of cases do not recover [26]. Therefore, due to the high mortality of patients with CS, it seems appropriate to intervene early in MI after evaluation by a multidisciplinary team with expertise in mitral valve disease.

## 5. Limitations

Even though this is a promising study, it should be emphasised that this is a retrospective study with a small sample size. For this reason, this registry is subject to selection bias, and the results should be considered with caution. First, regarding mortality, the outcomes are promising, and the mortality rates are lower than anticipated for this patient cohort. However, it is crucial to acknowledge the potential presence of indication bias. It is probable that only those patients deemed to be in a relatively healthier state were selected for the TEER procedure. Second, although it is true that the measurements were performed in all the centres following the recommendations of the current European guidelines for management of valvular heart disease, the analysis was not performed in a central laboratory, and there may be biases in the MR assessment. Therefore, the implantation success rate may differ between centres because it depends on the MR assessment. Third, the limited number of patients who received ECMO or IMPELLA precludes drawing definitive conclusions for this subgroup of patients. Fourth, although Kaplan–Meier survival curves showed positive outcomes in this study, the limited number of cases may lead to uncertain differences in the compared results. Therefore, these data should be interpreted with caution. For this reason, this study can only generate hypotheses. New prospective studies, such as the CAPITAL MINOS trial [27], will be necessary to evaluate the importance of TEER in CS, especially in those patients who require MCS.

## 6. Conclusions

The Mitra-ASSIST Registry describes the safety and feasibility of TEER therapies in patients with concomitant CS and MR requiring MCS. TEER in patients with concomitant CS and MR who require MCS would seem to be a promising therapeutic alternative with a high device procedural success rate and an acceptable mortality and HF admission rate at 12-month follow-up.

## Figures and Tables

**Table 1:** Baseline characteristics. Values are mean ± SD, n (%), or median (interquartile range). Abbreviations: ADA: anterior descending artery; AMI: acute myocardial infarction; CABG: coronary artery bypass grafting; CRRT: continuous renal replacement therapy; CX: circumflex artery; DCM: dilated cardiomyopathy; LM: left main artery; MCS: mechanical circulatory support; MV: mitral valve; NYHA: New York Heart Association; PCI: percutaneous coronary intervention; RCA: right coronary artery; STEMI: ST-elevation myocardial infarction; NSTEMI: non-ST-elevation myocardial infarction; STS: Society of Thoracic Surgeons.

	TOTAL (n = 24)
Age, years	65.7 ± 8.7
Female	17 (70.8)
Diabetes	12 (50)
Hypertension	13 (54.2)
Dyslipidemia	17 (70.8)
History of AMI	12 (50)
Prior PCI	10 (41.7)
Prior CABG	4 (16.7)
Any prior stroke	2 (8.3)
Peripheral vascular disease	7 (29.2)
STS risk score for MV repair	12.4 ± 10.1
EuroSCORE II risk score for MV repair	20.4 ± 17.8
Shock etiology	
STEMI	13 (54.2)
NSTEMI	5 (20.8)
Ischemic DCM	5 (20.8)
Non-ischemic DCM	1 (4.2%)
Culprit vessel	
LM	1 (4.2)
ADA	1 (4.2)
CX	10 (41.6)
RCA	6 (26)
SCAI stage	
C	13 (54.2)
D	11 (45.8)
INTERMACS profile	
1	6 (25.0)
2	7 (29.2)
3	11 (45.8)
Multiple organ failure at admission	16 (66.7)
Cardiac arrest (within 24 h)	1 (4.2)
Acute pulmonary edema	20 (83.3)
Intubated	11 (45.8)
CRRT	4 (16.7)
Admission to MCS (days)	5.9 ± 4.2

**Table 2:** Hemodynamics and laboratory data prior to the TEER procedure. Values are mean ± SD, n (%), or median (interquartile range). Abbreviations: eGFR: estimated glomerular filtration rate.

	TOTAL (n = 24)
Hemoglobin (g/dL)	10.4 (1.8)
eGFR (mL/min)	45.0 ± 19.4
Lactate, mmol/L	3.8 ± 2.6
Vasopressors support	18 (75)
Norepinephrine	16 (66.7)
Epinephrine	12 (50)
Inotropic support	15 (62.5)
Dobutamine	11 (45.8)
Levosimendan	4 (16.7)
Systolic blood pressure, mm Hg	97.5 ± 18.9
Diastolic blood pressure, mm Hg	63.5 ± 7.7
Mean arterial blood pressure, mm Hg	52.5 ± 8.2
Pulmonary capillary wedge pressure, mm Hg	22.5 ± 5.3
Pulmonary artery systolic pressure, mm Hg	57.4 ± 7.3

**Table 3:** Echocardiographic characteristics. Values are mean ± SD, n (%), or median (interquartile range). Abbreviations: ADA: anterior descendent artery; CX: circumflex artery; DCM: dilated cardiomyopathy; ECMO: extracorporeal membrane oxygenation; IABP: intra-aortic balloon pump; LM: left main artery; MCS: mechanical circulatory support; MR: mitral regurgitation; NSTEMI: non-acute ST-elevation myocardial infarction; RCA: right coronary artery; STEMI: acute ST-elevation myocardial infarction.

	TOTAL (n = 24)
LVEF (%)	33.4 ± 9.6
LVEDD (mm)	5.5 ± 0.9
TAPSE (mm)	17.4 ± 2.9
EROA (cm^2^)	0.7 ± 0.5
Mitral regurgitation: severity	
3+	1 (4.2)
4+	23 (95.8)
Mitral regurgitation: etiology	
Functional	20 (83.3)
Degenerative	4 (16.7)
De novo MR	19 (79.2)
Acute papillary muscle rupture	3 (12.5)

**Table 4:** Procedural features and in-hospital outcome. Values are mean ± SD, n (%), or median (interquartile range). Abbreviations: AKI: acute kidney injury; BARC: Bleeding Academic Research Consortium; ICU: intensive unit care; MCS: mechanical circulatory support; MV: mitral valve; MI: myocardial infarction.

	TOTAL (n = 24)
Admission to TEER (days)	8.8 ± 9.6
Procedural success	23 (95.8)
Procedural time (min)	95.6 (61–126)
No. of clips implanted	1.6 ± 0.8
Implanted clip size	
XT	6 (25)
NTW	5 (20.8)
NT	9 (37.5)
Post-procedural MV mean gradient (mmHg)	3.1 ± 1.2
MCS withdrawal	20 (83.3%)
Days until MCS withdrawal	1.7 ± 1.8
Bleeding	
BARC 3	2 (8.3)
BARC 4	1 (4.2)
AKI	7 (29.2)
MI	N/A
Stroke	N/A
ICU stay (days)	15 (5–20)
Intrahospital stay (days)	30 (17–36)
MR ≤ 2+ at discharge	21 (87.5)
Post-procedural intrahospital death	3 (12.5)

**Table 5:** Mechanical circulatory support features. Values are mean ± SD, n (%), or median (interquartile range). Abbreviations: ICU: intensive unit care; MCS: mechanical circulatory support; MR: mitral regurgitation; TEER: transcatheter edge-to-edge repair.

	IABP (n = 19)	IMPELLA (n = 2)	ECMO (n = 2)	ECMO/IMPELLA (n = 1)	*p* Value
Successful TEER procedure	18 (94.7)	2 (100.0)	2 (100.0)	1 (100.0)	0.96
Procedural time (min)	93.9 (30.0–180.0)	115.0 (90.0–140.0)	62 (45–115)	121.0 (N/A)	0.25
MCS withdrawal	16 (84.2)	2 (100)	2 (100)	N/A	0.09
ICU stay (days)	19.3 (23.0)	11.0 (8.5)	20.0 (22.6)	21.0 (N/A)	0.17
Intrahospital stay (days)	33.8 (29.8)	22.5 (14.8)	49.0 (38.2)	NA	0.85
Post-procedural MR ≤ 2+	19 (100)	2 (100)	2 (100)	1 (100)	0.83
Post-procedural intrahospital death	3.0 (15.8)	N/A	N/A	N/A	0.69

## Data Availability

The data presented in this study are available on request from the corresponding author.

## Appendix A

**Table A1:** Comparative analysis of mortality.

	ALIVE (n = 17)	DEATH (n = 7)	TOTAL (n = 24)	*p* Value
Age, years	62.5 ± 7.7	73.6 ± 5.7	65.7 ± 8.7	0.002
Female	12 (70.6)	5 (71.4)	17 (70.8)	
Diabetes	8 (47.1)	4 (57.1)	12 (50.0)	0.653
Prior PCI	7 (41.2)	3 (42.9)	10 (41.7)	0.939
INTERMACS profile				0.575
1	5 (29.4)	1 (14.3)	6 (25.0)	
2	4 (23.5)	3 (42.9)	7 (29.2)	
3	8 (47.1)	3 (42.9)	11 (45.8)	
SCAI stage				0.380
C	14 (82.4)	4 (57.1)	18 (75)	
D	3 (17.6)	3 (42.9)	6 (25.0)	
STEMI	10 (58.8)	3 (42.9)	13 (54.2)	0.476
Mitral regurgitation: severity				0.512
3+	1 (5.9)	0	1 (4.2)	
4+	16 (94.1)	7 (100.0)	23 (95.8)	
Mitral regurgitation: etiology				
Functional	15 (88.2)	5 (71.4)	20 (83.3)	
Degenerative	2 (11.8)	2 (28.6)	4 (16.7)	
Acute papillary muscle rupture	2 (11.8)	1 (14.3)	3 (12.5)	0.865
LVEF (%)	33.0 ± 9.3	34.4 ± 10.9	33.4 ± 9.6	0.742
Admission to TEER (days)	8.6 ± 9.0	9.1 ± 11.7	8.8 ± 9.6	0.901
